# The oligodendrocyte-enriched orphan G protein-coupled receptor Gpr62 is dispensable for central nervous system myelination

**DOI:** 10.1186/s13064-021-00156-y

**Published:** 2021-11-29

**Authors:** Curtis M. Hay, Stacey Jackson, Stanislaw Mitew, Daniel J. Scott, Matthias Koenning, AeSoon L. Bensen, Helena Bujalka, Trevor J. Kilpatrick, Ben Emery

**Affiliations:** 1 Department of Anatomy and Neuroscience, University of Melbourne, Melbourne, Victoria Australia; 2 Present address: BC Children’s Hospital, Vancouver, British Columbia Canada; 3 Florey Institute of Neuroscience and Mental Health, Parkville, Victoria Australia; 4 Present address: Duke-NUS Medical School, Singapore, Singapore; 5 Department of Biochemistry and Pharmacology, The University of Melbourne, Melbourne, Victoria Australia; 6 Present address: Center for Precision Environmental Health at Baylor College of Medicine, Houston, Texas USA; 7 Jungers Center for Neurosciences Research, Department of Neurology, Oregon Health & Science University, Portland, Oregon USA

**Keywords:** CNS myelination, Oligodendrocyte, Glia, Myelin sheath, Axoglial interactions, Gpr62, Mouse genetics

## Abstract

**Background:**

Myelination is a highly regulated process in the vertebrate central nervous system (CNS) whereby oligodendrocytes wrap axons with multiple layers of insulating myelin in order to allow rapid electrical conduction. Establishing the proper pattern of myelin in neural circuits requires communicative axo-glial interactions, however, the molecular interactions that occur between oligodendrocytes and axons during developmental myelination and myelin maintenance remain to be fully elucidated. Our previous work identified G protein-coupled receptor 62 (Gpr62), an uncharacterized orphan g-protein coupled receptor, as being selectively expressed by mature oligodendrocytes within the CNS, suggesting a potential role in myelination or axoglial interactions. However, no studies to date have assessed the functional requirement for Gpr62 in oligodendrocyte development or CNS myelination.

**Methods:**

To address this, we generated a knockout mouse strain lacking the *Gpr62* gene. We assessed CNS myelination during both postnatal development and adulthood using immunohistochemistry, electron microscopy and western blot. In addition, we utilized AAV-mediated expression of a tagged Gpr62 in oligodendrocytes to determine the subcellular localization of the protein in vivo.

**Results:**

We find that virally expressed Gpr62 protein is selectively expressed on the adaxonal myelin layer, suggestive of a potential role for Gpr62 in axo-myelinic signaling. Nevertheless, *Gpr62* knockout mice display normal oligodendrocyte numbers and apparently normal myelination within the CNS during both postnatal development and adulthood.

**Conclusions:**

We conclude that in spite of being well-placed to mediate neuronal-oligodendrocyte communications, Gpr62 is overall dispensable for CNS myelination.

## Background

Myelination of axons has evolved as a mechanism to allow for rapid, energy efficient conduction of nerve impulses [1]. Within the central nervous system (CNS) of jawed vertebrates, this myelination is carried out by oligodendrocytes, each of which can ensheath 30–50 nearby axons with multilayered wraps of myelin [2]. The presence of this myelin and the clustering of ion channels at the nodes of Ranvier allow for the process of saltatory conduction, dramatically increasing conduction velocities for a given axonal diameter. In addition, oligodendrocytes provide axons with trophic and metabolic support [3–8]. Accordingly, when myelin or oligodendrocytes are disrupted, axons can lose their ability to properly transmit electrical signals and eventually degenerate, sometimes leading to significant and debilitating neurological deficits. During both developmental and adult myelination, resident oligodendrocyte progenitor cells (OPCs) proliferate, differentiate into postmitotic oligodendrocytes, contact multiple nearby axons and finally commit to myelinating a subset of these axons [9]. Although much of this myelination process appears to be developmentally hardwired, there is increasing evidence that neuronal activity can modulate myelination, perhaps representing an aspect of neuroplasticity [10, 11]. Although once established myelin becomes relatively stable, there is evidence that even mature myelin sheaths may be capable of some degree of remodelling [12–15].

Fundamental to understanding both developmental myelination and myelin plasticity is elucidation of the signals that neurons and other CNS cell types provide to regulate OPC and oligodendrocyte behaviour. Perhaps not surprisingly given their role in mediating a wide range of cell-cell communication, G protein-coupled receptors (GPCRs) have been shown to play a pivotal role in regulating numerous aspects of myelination (reviewed in [16]). For example, Gpr17 is expressed in OPCs and pre-myelinating oligodendrocytes and acts to inhibit their differentiation; knockouts display precocious myelination and agonists inhibit oligodendrocyte differentiation in culture [17, 18]. Gpr37, which is expressed at later stages of the lineage, appears to play a similar role in limiting myelination [19]. Both Gpr17 and Gpr37 can signal through Gα_i/o_ proteins [18–20], indicating that these two receptors play sequential roles in preventing excessive or premature myelination via a cAMP-dependent mechanism. The adhesion GPCR Gpr56 also prevents precocious OPC differentiation; mouse and fish mutants for Gpr56 show enhanced OPC differentiation and reduced proliferation at early ages, leading to a subsequent depletion of OPCs and myelinating cells at later stages of development [21–23].

In contrast to Gpr17, Gpr37 and Gpr56, all which largely act to inhibit myelination during early oligodendrocyte development, less is known about the GPCRs that may act within mature, myelinating oligodendrocytes to mediate axoglial interactions or oligodendrocyte maintenance. Our previous work, in which we transcriptionally profiled glial and neuronal populations in the mouse brain, identified the *Gpr62* transcript as one of the most oligodendrocyte-enriched transcripts in the brain [24]. *Gpr62* transcripts showed an equivalent enrichment in myelinating oligodendrocytes as transcripts coding for established myelin proteins such as Myelin oligodendrocyte glycoprotein (MOG), Myelin basic protein (MBP) and Proteolipid protein (PLP), and appeared to be only expressed at mature (MOG+) stages of the lineage. This distinguishes Gpr62 from other GPCRs with known roles at earlier stages of differentiation. *Gpr62* is expressed in the brain and testes, but appears to show very limited expression elsewhere in the body [25, 26]. An orphan GPCR, Gpr62 and the related Gpr61 are classified as class A (rhodopsin-like) GPCRs [27]. They are structurally related to the serotonin 5-HT receptor [26], however, direct binding of biogenic amines to Gpr62 or Gpr61 has not been demonstrated. A recent report indicates that both Gpr61 and Gpr62 form complexes with the melatonin MT2 receptor and modulate its activity, but show no binding affinity for melatonin themselves [28]. As such the relevant endogenous ligands for Gpr62 and its biological functions remain unknown.

In light of the selective expression of Gpr62 in myelinating oligodendrocytes, we investigated both the subcellular localization of Gpr62 protein and its role in regulating CNS myelination using *Gpr62* null mice. We find that Gpr62 protein is expressed along the axonal-adjacent, or adaxonal side of myelin internodes, making it an excellent candidate for mediating axon-oligodendrocyte communications. Nevertheless, *Gpr62* knockout mice have no overt behavioral phenotype, show normal numbers of oligodendrocytes and structurally normal myelin. Although a role for Gpr62 in mediating more subtle aspects of myelin or axoglial communications cannot be ruled out, these results suggest that Gpr62 has either a minor or a redundant role in regulating CNS myelination.

## Materials and methods

### Generation of Gpr62 knockout mice

*Gpr62* floxed and knockout mice were generated by cloning a region encompassing the coding region and most of the 3′ untranslated region (UTR) of mouse *Gpr62* (mm10 chr9:106,464,426-106,466,226) into the pEZ-Frt-lox-DT targeting vector (kind gift of Prof. Klaus Rajewsky, Addgene 11,736). A 5 kb 5′ arm and a 3 kb 3′ arm was cloned into the NotI and the XhoI site, respectively, to enable targeting of the construct via homologous recombination into the E14 embryonic cell (ES) line. The ES cells were screened using Southern blots for homologous recombination of the NeoR-loxP-*Gpr62*-loxP allele into the genome. An appropriately targeted clone was injected into C57BL/6 N embryos to give rise to a chimeric founder and subsequent germline transmission of the NeoR-loxP-Gpr62-loxP allele. To remove the Frt-flanked neomycin resistance cassette, the NeoR-loxP-Gpr62-loxP mice were crossed onto the FlpER strain [29], generating a *Gpr62*^Floxed^ line. This line was subsequently crossed onto a ubiquitous heat-shock Meox2-Cre line [30] to drive germ-line deletion of *Gpr62* and generation of a knockout allele (*Gpr62*^KO^). Knockout mice were genotyped using the following primers. Common upper: AAGGGGTGGTGCTAATGATG wild-type lower: CCAGGGAGTGGTCATGAGTT knockout lower: TACCGGCTGTCACTCTGATG. Mice were maintained on a mixed C57BL/6 N × 129 background (predominantly C57BL/6 N). All experiments used knockout and wild-type littermates generated from heterozygous crosses.

### Animal handling

All mice were housed within the animal facility at the Melbourne Brain Centre (University of Melbourne) and the Oregon Health & Science University under specific pathogen free conditions, a 12-h light/dark cycle and with free access to food and water. Mice were sacrificed at the indicated ages by deep anesthesia with ketamine xylazine cocktail (400 mg/kg ketamine and 60 mg/kg xylazine i.p.) followed by transcardial perfusion with either phosphate buffered saline/PBS (for biochemistry) or PBS followed by 4% paraformaldehyde in PBS (for histology).

### Immunohistochemistry and histology

Following perfusion, spinal cords, brains, and optic nerves were resected and postfixed in 4% PFA for 2 h, followed by 24 h in 30% sucrose in PBS at 4 °C. Tissue was then mounted in OCT (TissueTek) for cryosectioning of 10 μm sections. Sections (longitudinal optic nerve sections or transverse sections through the spinal cord at the lumbar expansion) were blocked and stained with 10% fetal bovine serum with 0.3% Triton-X overnight using the following antibodies: rat anti-MBP (Millipore MAB386), mouse CC1 monoclonal (Millipore OP80), rabbit anti-Chondroitin sulfate proteoglycan NG2 (Millipore AB5320), mouse anti-MAG (Millipore MAB1567), chick anti-GFP (AbCam 13,970), rabbit anti-Flag (Cell Signaling 2368), rabbit anti-Nav1.6 (Alomone labs ASC-009) or mouse anti-Caspr (NeuroMab K65/35). Sections were then incubated for 2 h with their respective fluorescent secondary antibodies (ThermoFisher highly cross-absorbed AlexaFluor conjugated secondaries diluted 1:500 in PBS) and mounted using Prolong Gold Anti-Fade reagent with DAPI (ThermoFisher). Cell counts were performed using images taken from three to six cryosections per animal, with 100 μm or greater distance between each section, using 20x objective on a Zeiss Axioplan microscope (Zeiss, Thornwood, NY, USA) with an AxioHRc camera. All cell counts were analyzed using NIH ImageJ software [31] on blinded images, and mean densities were calculated for each animal. Images for assessment of Gpr62-Flag co-localization with MBP and MAG were captured using a Zeiss LSM 980 Laser-Scanning Confocal with Airyscan 2. Black Gold staining was performed on 50 μm vibratome sections of the brain (Bregma − 2.0 mm) using the Black-Gold II staining kit (EMD Millipore AG400) as per manufacturer’s protocol with the exception that the development step was performed for 4–5 h at room temperature. Black Gold-stained sections were imaged on a Zeiss Axioplan microscope at × 10 magnification.

### In situ RNA hybridization

Spinal cords and optic nerves from *Gpr62* knockout and wildtype mice were fixed in 4% PFA for 2 h and sunk in 30% sucrose (PBS). Digoxigenin (DIG)-labeled antisense probes for *Gpr62* were generated using a plasmid encoding the coding sequence of mouse *Gpr62* and the DIG RNA Labeling kit (Roche, Germany) following the manufacturers protocol. In-situ hybridization was performed using a standard protocol as previously described [24].

### Ultrastructural analysis of myelin

Optic nerves, sections of the thoracic spinal cord and rostral sections of the corpus callosum were post-fixed in 4% PFA, 2.5% glutaraldehyde in 0.1 M cacodylate buffer for 24 h prior to embedding in epoxy resin. The resin-embedded tissues were oriented to analyze axonal cross-sections and cut into semi-thin (0.5 μm) sections and stained with toluidine blue to visualize the structural quality of the tissue before ultra-thin (90 nm) diamond knife sectioning. Ultra-thin sections were imaged using a Siemens Stereoskop Transmission Electron Microscope (TEM) at magnifications of 3000x and 10,000x. Three animals per genotype in each time point were analyzed for the number of myelinated axons and for myelin thickness (g-ratios). The percent of axons myelinated were assessed using Image J [31], while the g-ratios were measured using Imagetrak (image analysis software written by Peter K. Stys, https://stysneurolab.org/imagetrak/). G-ratios (axonal diameter/external myelin diameter) were calculated by measuring the area of the myelin and of the axon and then converting areas to diameters for the g-ratio calculations, with 200–300 myelinated axons being assessed per animal for g-ratios. Over 300 axons were analyzed to calculate the proportion of myelinated axons per animal using NIH Image J software, with all axons within an image being classified as myelinated or nonmyelinated.

### qRT-PCR

Optic nerves were extracted and processed with Qiagen RNeasy tissue mini kits for total RNA as per the manufacture’s protocol. Reverse transcription of RNA was performed with 1 μg of RNA and oligo(dT) primers using the Taqman Reverse Transcription (RT) kit (Applied Biosystems). Products of the RT reactions were used for quantitative real-time PCR (qPCR) using an ABI7700 sequence detection system (Applied Biosystems). The 2^-ΔΔCT^ method was used to assess relative expression from amplified RNA samples, with values normalized to 18S ribosomal subunit expression. Primer3 (National Center for Biotechnology Information) and qPrimerDepot (primerdepot.nci.nih.gov) were used to design the qPCR primers for *Gpr62* (TTTATCCTGGCGGTTCTCGTA and TGCGCTAAGTAGAAGGCATCTTG) and *18S* (CGGCTACCACATCCAAGGAA and GCTGGAATTACCGCGGCT).

### Western blotting

Whole brains were extracted from PBS-perfused *Gpr62* wild-type and knockout mice and dounce homogenized in RIPA buffer (50 mM Tris pH 8.0, 150 mM NaCl, 1% NP-40, 0.5% Sodium deoxycholate, 0.1% SDS, 1 mM EDTA and 0.5 mM EGTA) with complete protease inhibitors (Roche). Lysates were clarified at 21,500 x g at 4 °C for 10 min and the supernatants diluted with Laemmli Sample Buffer. Twenty microgram of protein per sample was run on NuPage Bis-Tris gels (ThermoFisher) and transferred to PVDF (Millipore). Blots were blocked in 5% skim milk powder in tris buffered saline with 0.1% Tween-20 and probed with mouse anti-beta-tubulin (Developmental Studies Hybridoma Bank E7), anti-MBP (Millipore MAB386) or anti-PLP (clone AA3 [32]) and appropriate HRP-conjugated secondaries (Cell Signaling). Blots were imaged on a G:Box gel imaging system (Syngene) and densitometry analysis performed using Image J software [31].

### AAV expression

The pAAV-pMBP-GFP construct, containing the coding region of eGFP behind the 1.94 kb *Mbp* promoter [33], was a kind gift of Prof. Matthias Klugmann (University of New South Wales). pAAV-pMBP-Gpr62-Flag was generated by excising the coding sequence of eGFP and replacing it with the coding region of mouse Gpr62 with a C-terminal Flag tag. The AAV-pMBP-eGFP and AAV-pMBP-Gpr62-Flag vectors were packaged as AAV2 by the Molecular Virology Core, Oregon National Primate Center, as previously described [34] and diluted to 2 × 10^12^ vg/ml. C57BL/6 N pups were cryo-anesthetized at postnatal day 6 and 1 μl of packaged AAV was stereotaxically injected using a 34G beveled needle attached to a Hamilton syringe at Lambda coordinates AP + 3.2, ML -1.3, DV − 1.5 (broadly corresponding to the region of the developing corpus callosum). Once injected, pups were re-warmed on a heating pad until ambulatory and returned to the home cage.

### Statistical analysis

For cell counts, percent axons myelinated and g-ratio analyses, the mean and standard error of the mean (SEM) for each experimental group were calculated using the mean values for each animal. Two-way ANOVAs with Bonferroni’s *post-hoc* tests were used to statistically analyze the data using Graphpad Prism software. All data are depicted as mean ± SEM.

## Results

### Gpr62 is highly enriched in mature oligodendrocytes in the mature CNS and localizes along the myelin internode

**Fig. 1:**
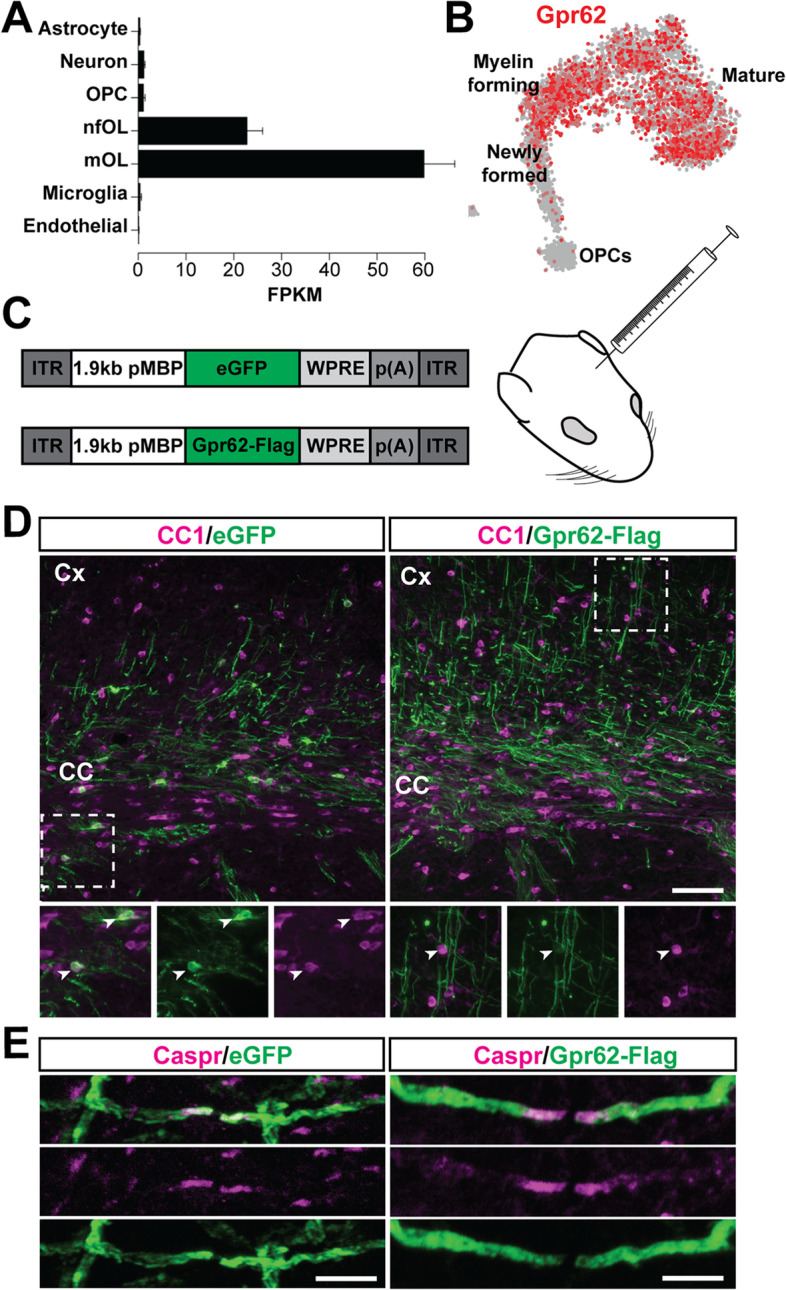
Gpr62 expression is highly enriched in mature oligodendrocytes, where the protein is localized along the myelin sheath. **A** Expression of *Gpr62* in astrocytes, neurons, OPCs, newly formed oligodendrocytes, myelinating oligodendrocytes, microglia and brain endothelial cells acutely purified from the postnatal mouse brain. Expression data from [35] nfOL: Newly-formed oligodendrocyte. mOL: Myelinating oligodendrocyte. (B) Single cell RNA-Seq indicates that *Gpr62* expression is restricted to myelin forming and mature oligodendrocytes. Expression data and t-SNE plot of oligodendroglia from [36]. (C) Design of AAV expression constructs to express eGFP or Gpr62-Flag behind 1.9 kb of the *Mbp* promoter. AAVs were injected into the corpus callosum and striatum of P6 mice. (D) AAV-mediated expression of eGFP or Gpr62-Flag in the infected corpus callosum and overlying cortex. As previously published [33, 37], the 1.9 kb Mbp promoter strongly drove eGFP expression in mature (CC1+) oligodendrocytes. Gpr62-Flag expression was faint or absent in oligodendrocyte cell bodies, but readily detectable along myelin internodes. Scale bars: 50 μm. (E) Both eGFP and Gpr62-Flag expressing myelin internodes were flanked by Caspr+ paranodes, confirming oligodendroglial rather than axonal expression of the AAV constructs. Scale bars: 5 μm

In a previous Affymetrix study on purified cell types from the mouse CNS we identified *Gpr62* as being highly enriched in mature (MOG+) oligodendrocytes. We subsequently confirmed via in situ hybridization that the distribution of *Gpr62* expressing cells in the brain matched the expected pattern for mature oligodendrocytes [24]. This high-enrichment of *Gpr62* expression in myelinating oligodendrocytes has subsequently been confirmed by both bulk and single cell RNA-Seq experiments [35, 36], both of which identify strongest expression of *Gpr62* in the mature oligodendrocyte populations (Fig. 1A, B) and little or no expression in OPCs or other CNS populations.

Given this preferential oligodendrocyte expression, we sought to determine the subcellular localization of Gpr62 in order to provide potential insight into its likely signaling location. Few commercial antibodies are available for Gpr62 and we were unable to identify any that specifically detected Gpr62 (tested by immunohistochemistry and western blot validation in *Gpr62* knockout tissue; data not shown). We therefore generated an AAV-pMBP-Gpr62-Flag construct to express a tagged variant of Gpr62 in myelinating oligodendrocytes in vivo (Fig. 1C). Previous characterization of this AAV construct containing 1.9 kb of the murine *Mbp* promoter shows that it strongly and specifically drives expression of genes to postmitotic oligodendrocytes when injected into the developing mouse brain [33, 38]. Thirty days after injection of AAV-pMBP-Gpr62-Flag or AAV-pMBP-eGFP into the developing corpus callosum and striatum at postnatal day 6, expression of Flag or eGFP was readily detectable in the region of AAV injection. As previously reported [33], expression of eGFP was essentially restricted to CC1+ oligodendrocytes in the AAV-pMBP-eGFP condition, confirming specificity of the promoter (Fig. 1D). Unexpectedly, there was remarkably little expression of the Gpr62-Flag on CC1^+^ oligodendrocyte cell bodies, though occasional faint Flag expression could be seen along the membrane of CC1^+^ infected cells. Rather, strong expression of Flag was evident along myelinated axons within the region of infection. Expression of both the AAV-pMBP-eGFP and AAV-pMBP-Gpr62-Flag constructs were found in discrete segments flanked by Caspr staining (Fig. 1E), confirming that the AAV-induced expression was present along myelin internodes of infected oligodendrocytes rather than resulting from off-target AAV expression in the neuronal population. Consistent with its cytoplasmic localization, the virally expressed eGFP was somewhat enriched at the non-compact myelin at the paranode; the Gpr62-Flag expression was typically evenly distributed along the myelin internodes. Given other transmembrane proteins (e.g., membrane-associated GFP) do not so strikingly localize to the myelin sheath preferentially over the cell membrane of the oligodendrocyte cell body [39, 40], this strongly suggests that the Gpr62 protein contains sequences to target it to the myelin sheath.

**Fig. 2:**
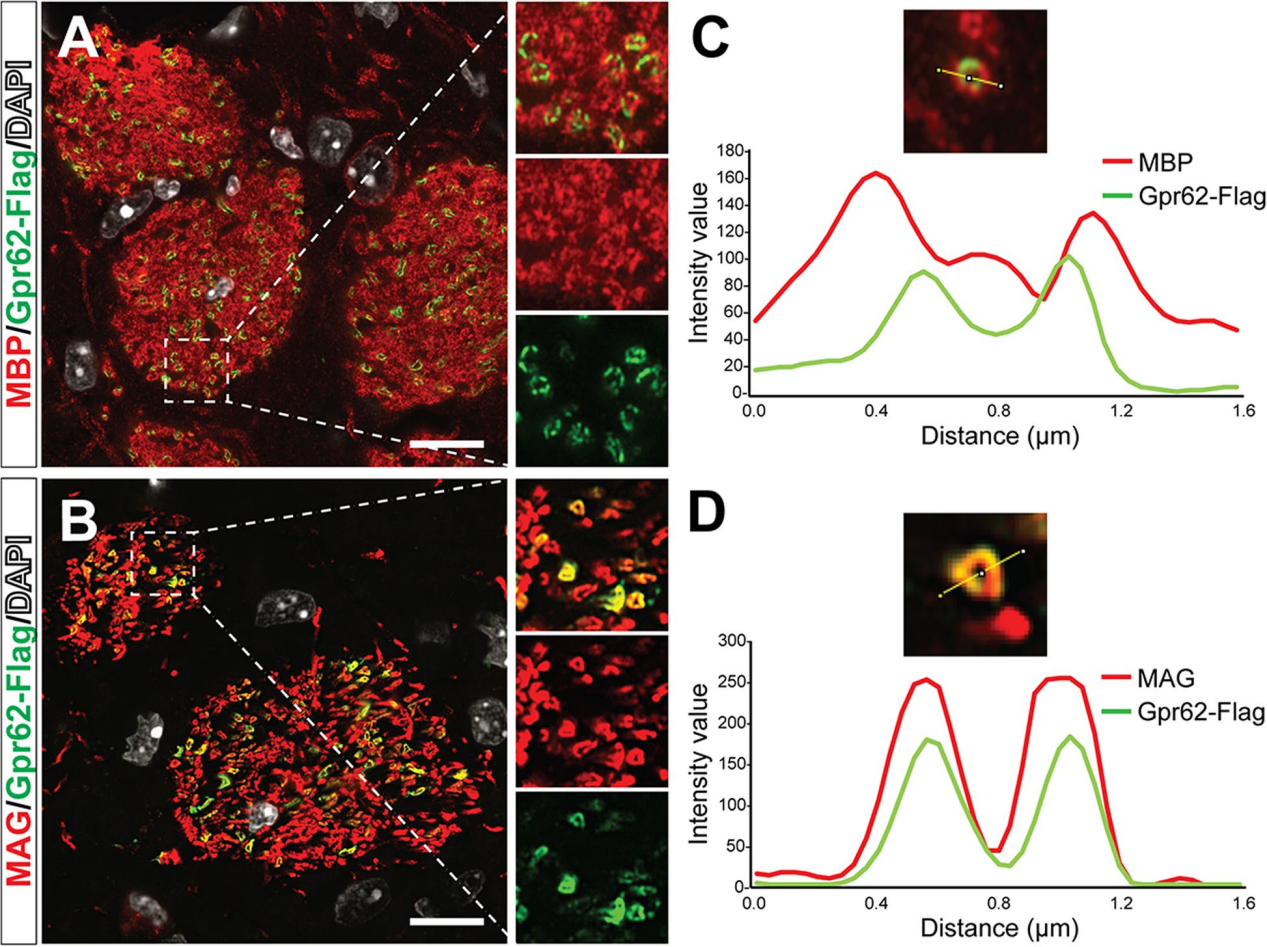
Gpr62-Flag expression is localized to the adaxonal myelin surface. **A, B** AAV-pMBP-Gpr62 expression in the striatum with expressing myelin sheaths caught in cross section. Sections were co-stained with anti-MBP (compact myelin, **A**) or anti-MAG (adaxonal myelin and inner tongue, **B**). **C-D** Profile of staining intensity for Gpr62-Flag, MBP and MAG in representative myelin segments. Gpr62-Flag expression in a myelin sheath was typically internal to the majority of MBP staining (**C**), but showed a near perfect co-localization with MAG (**D**), consistent with an adaxonal localization of Gpr62-Flag protein. Scale bars: 10 μm

Presumably, the expression of the Gpr62 protein could be along the adaxonal side, putting it in close proximity to the myelinated axons, and/or the abaxonal side, facing the extracellular matrix. To investigate whether the tagged Gpr62 showed a preference, we co-stained sections of AAV-pMBP-Gpr62-Flag infected brains with anti-Flag and either anti-MBP (present throughout the compact myelin) or anti-myelin-associated glycoprotein (MAG, enriched at the innermost, adaxonal myelin membrane where it contacts the axon) [41]. We imaged coronal sections of the corpus striata of infected mice using super-resolution microscopy to image Gpr62-Flag^+^ myelin internodes caught in cross section (Fig. 2A, B). The anti-Flag signal showed poor co-localization with MBP, with Gpr62-Flag signal in infected myelin internodes typically being surrounded by a ring of MBP staining (Fig. 2A, C). In contrast, Gpr62-Flag staining showed almost perfect co-localization with MAG (Fig. 2B, D). Given the tight spacing between myelin layers largely excludes bulky transmembrane proteins from the compact myelin [42], it therefore seems likely that the virally expressed, and presumably also endogenously expressed, Gpr62 is localized along the innermost myelin wrap in close proximity to the axon.

### Gpr62 is not required for oligodendrocyte differentiation or survival

**Fig. 3:**
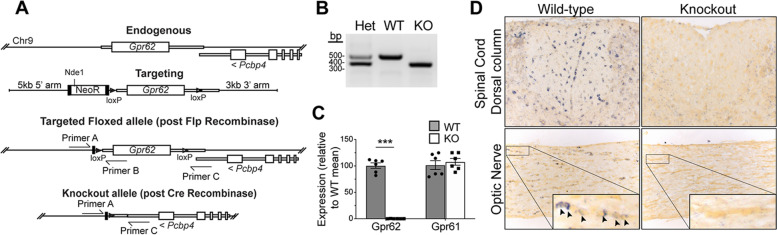
Generation of *Gpr62* knockout mice. **A** Schematic of conventional targeting strategy used to generate a *Gpr62* knockout mouse line. Gpr62 is encoded by a single exon gene with its 3′ UTR overlapping the 3′ UTR of *Pcbp4* on the opposite strand. A targeting vector was designed to insert loxP sites upstream and within the *Gpr62* 3′ UTR. Correctly targeted mice were crossed to FlpER and germline expressing Cre lines to generate the loxP flanked and knockout mouse lines, respectively. **B** PCR confirmation of generation of heterozygous and knockout mice, using the primers in (**A**). **C** qPCR expression of *Gpr62* and *Gpr61* expression in the brains of 8-week old control and knockouts (*n* = 6). *Gpr62* null mice showed the expected loss of *Gpr62* mRNA expression without any detectable compensatory upregulation of *Gpr61.* **D**. *Gpr62* expression can be seen in chains of glial cells (presumptive oligodendrocytes) in the white matter of the spinal cord and the optic nerve of the wild-type littermates, with this expression absent in the knockout (inserts, **D**)

In order to assess the role of Gpr62 in myelination, we used homologous recombination in ES cells to generate a *Gpr62* null allele. Gpr62 is encoded by a single-exon gene; because its 3′ UTR region overlaps with the 3′ untranslated region of the Poly(RC) Binding Protein 4 (*Pcbp4*) gene on the opposite strand of chromosome 9, the knockout allele was designed to delete the entire coding region of *Gpr62* and the majority of the 3′ UTR, leaving the region of overlap with *Pcbp4* intact (Fig. 3A). We first used homologous recombination in ES cells to generate a loxP flanked *Gpr62* allele, successfully obtaining germline mice containing the Gpr62^Floxed-Neo^ allele. These mice were sequentially crossed to FlpER [29] and Meox2-Cre mice [30] to obtain first *Gpr62*^Floxed^ and subsequently *Gpr62*^KO^ mice. As preliminary results indicated the *Gpr62*^KO/ KO^ mice were viable (Fig. 3B), we elected to first assess the full knockout line rather than conditional knockout mice.

A recent report investigating the role of Gpr62 in spermatogenesis reported that *Gpr62* null mice are viable and without an overt phenotype [25]. Consistent with this report, we found that *Gpr62*^KO/ KO^ mice were generated at the expected Mendelian ratios from heterozygous crosses (Table 1). *Gpr62*^KO/ KO^ mice and were indistinguishable from their *Gpr62*^WT/KO^ or *Gpr62*^WT/WT^ littermates in both weight and apparent health, with no overt phenotype and viability to at least 1 year of age (data not shown). We confirmed the loss of *Gpr62* expression in the knockout mice using qRT-PCR and in situ hybridization using a probe corresponding to the coding region of *Gpr62*. *Gpr62*^KO/ KO^ mice showed no detectable *Gpr62* expression within the brain by qPCR, and the normal expression of *Gpr62* transcript in spinal cord and optic nerve oligodendrocytes was absent by in situ hybridization (Fig. 3C, D). There was no detectable change in the expression of the closely related *Gpr61* gene in the brains of *Gpr62*^KO/ KO^ mice relative to wild-type littermates at 8 weeks of age, indicating a lack of compensatory up-regulation (Fig. 3C)*.*

**Table 1:** Gpr62^KO/KO^ mice are born at expected Mendelian ratios from heterozygous crosses

WT/WT		WT/KO		KO/KO			
Observed	Expected	Observed	Expected	Observed	Expected	χ^2^	*p*-value (df = 2)
88	93	193	186	91	93	0.575	0.75

Observed numbers of *Gpr62*^WT/WT^, *Gpr62*^WT/KO^ and *Gpr62*^KO/KO^ progeny resulting from heterozygous breeding pairs. The distribution of genotypes does not significantly differ from expected Mendelian ratios (*p* > 0.05 by chi-square test)

**Fig. 4:**
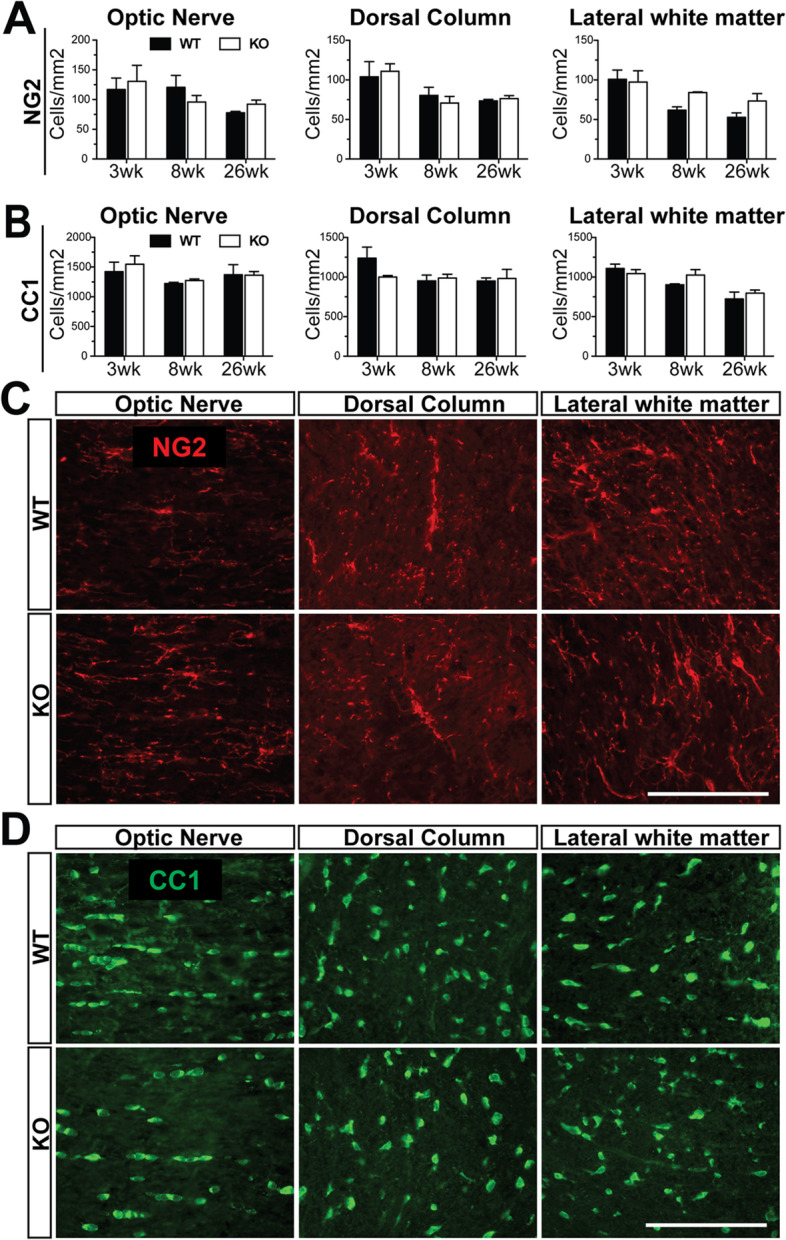
Loss of Gpr62 does not alter the density of OPCs or oligodendrocytes. **A** Quantification of the density of NG2+ OPCs and CC1+ oligodendrocytes in the optic nerve, dorsal column of the spinal cord and lateral white matter of the spinal cord of *Gpr62*^WT/WT^ and *Gpr62*^KO/KO^ mice at 3 weeks, 8 weeks and 26 weeks of age. *n* = 3 per condition. **C, D** Representative images of NG2 (**C**) and CC1 (**D**) staining at 8 weeks of age in each area in *Gpr62*^WT/WT^ and *Gpr62*^KO/KO^ animals. Scale bars: 100 μm

To determine whether deletion of *Gpr62* caused an alteration in oligodendrocyte development or survival, we assessed densities of OPCs and mature oligodendrocytes in several regions of the CNS (optic nerve and the dorsal column and lateral white matter of the spinal cord). Consistent with the lack of detectable *Gpr62* expression within OPCs, no alteration was seen in the density of NG2^+^ OPCs at any age assessed (3 weeks, 8 weeks or 26 weeks postnatal, *n* = 3, *p* > 0.05 for all time-points) (Fig. 4A, C). To determine whether the absence of Gpr62 altered the generation or survival of mature oligodendrocytes, we assessed the same regions of the CNS for expression of the mature oligodendrocyte marker CC1. We found no difference in the density of CC1^+^ oligodendrocytes in either the spinal cord white matter tracts or the optic nerve at ages corresponding to active myelination (3 weeks), established myelin (8 weeks) or adulthood (26 weeks) (Fig. 4B, D, *n* = 3, *p* > 0.05 for all time-points). This suggests that Gpr62 is not required either for successful oligodendrocyte differentiation or survival into adulthood.

### Loss of Gpr62 is dispensable for CNS myelination

**Fig. 5:**
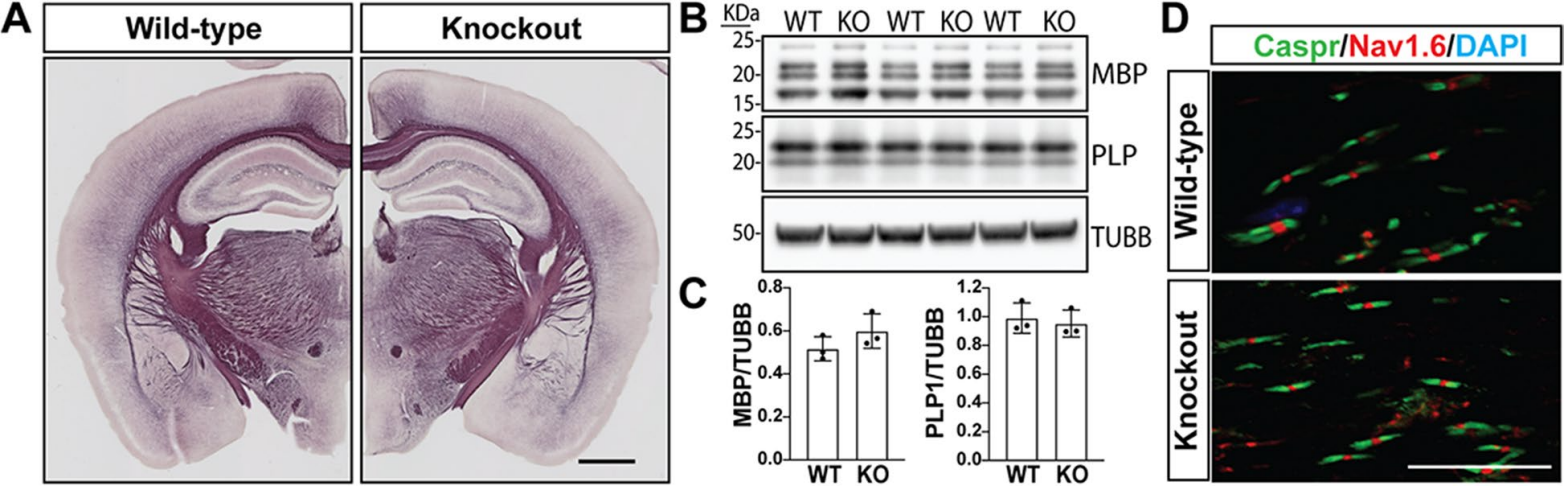
Loss of Gpr62 does not grossly disrupt myelin formation in the brain. **A** Representative black gold staining of coronal brain hemisections of 8-week old *Gpr62*^WT/WT^ and *Gpr62*^KO/KO^ mice. The overall intensity and distribution of black gold stained myelin is similar between each genotype. Scale bar: 1 mm. **B** Western blot analysis of myelin proteins MBP and PLP in whole brain lysates from 8-week old Gpr62 wild-type and knockouts. Quantified in (**C**) relative to beta tubulin (TUBB) as a control for protein loading (*p* > 0.05 for both comparisons). **D** Representative images of the optic nerves of an 8-week old *Gpr62*^WT/WT^ and *Gpr62*^KO/KO^ mouse stained with the paranodal marker Caspr and nodal marker Nav1.6. Scale bar: 10 μm

*Gpr62* expression is induced late in oligodendrocyte differentiation (Fig. 1B), suggesting that even if it is dispensable for oligodendrocyte differentiation and the early stages of myelination it may have roles in regulating later stages of the myelination process. We therefore assessed the extent of myelination in *Gpr62* null mice and control littermates at both the gross and ultrastructural level. Black gold-stained brain sections from 8-week old *Gpr62*^WT/WT^ and *Gpr62*^KO/KO^ mice revealed no gross alterations in either the distribution or intensity of myelin staining in the brains of Gpr62^KO/KO^ mice relative to their wild-type littermates (Fig. 5A). For a quantifiable assessment of the degree of overall CNS myelination we measured the expression of two key myelin proteins, PLP and MBP, in whole brain lysates at 8 weeks of age. There was no statistical difference in the abundance of these proteins in whole brain lysates from *Gpr62*^KO/KO^ mice relative to their wild-type littermates (Fig. 5B, C), again indicating that the loss of Gpr62 did not alter the total degree of myelin in the adult CNS. Staining for the nodal marker Nav1.6 and paranodal marker Caspr were also comparable between the optic nerves of *Gpr62*^WT/WT^ and *Gpr62*^KO/KO^ mice at 8 weeks of age, suggesting that loss of Gpr62 did not grossly alter the morphology of the nodes of Ranvier (Fig. 5D).

**Fig. 6:**
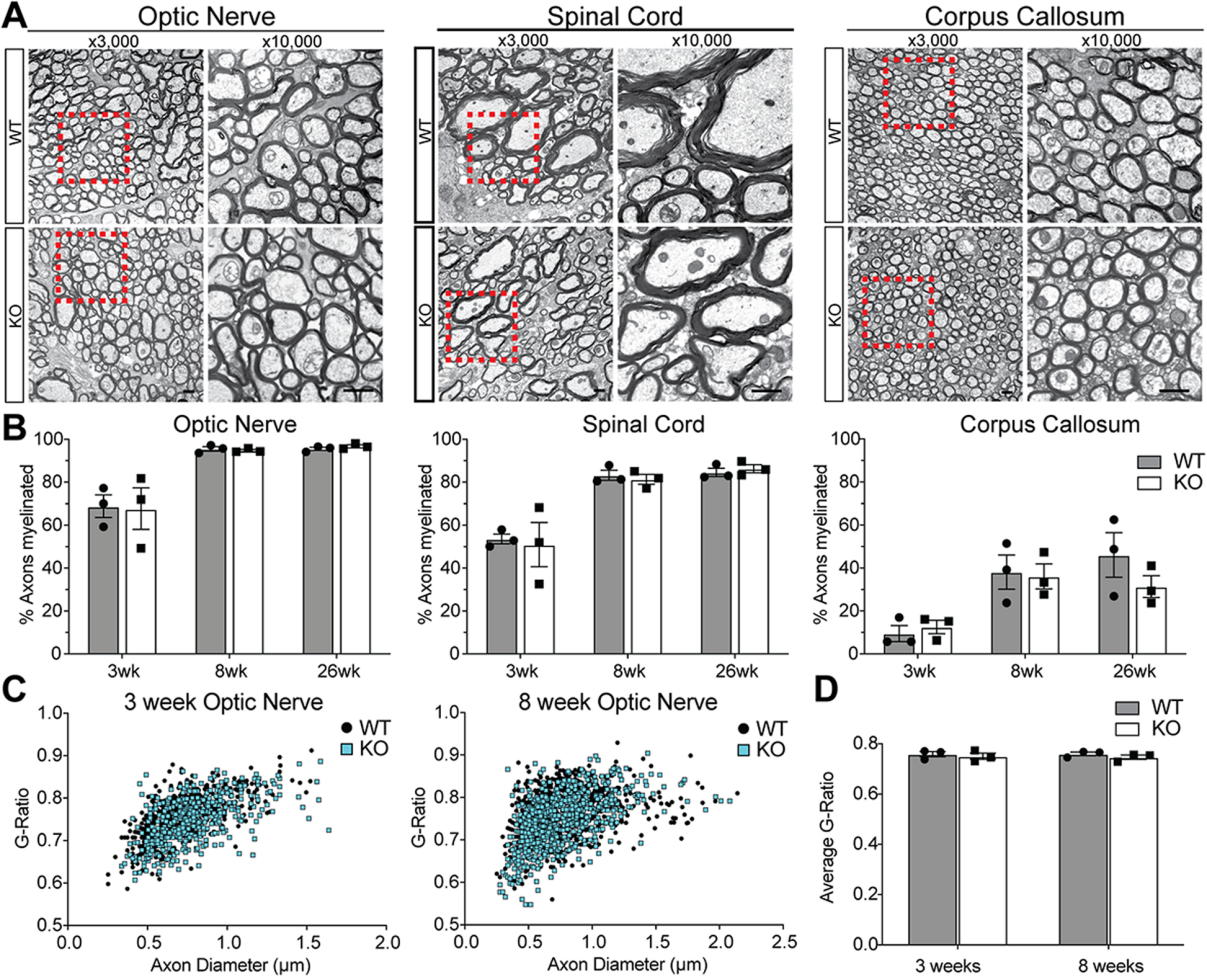
Loss of Gpr62 does not grossly alter myelin thickness or extent in the CNS. **A** Representative transmission electron microscopy images of myelinated axons in the optic nerve, spinal cord and corpus callosum at 8 weeks of age in *Gpr62*^WT/WT^ and *Gpr62*^KO/KO^ mice. Scale bars: 1 μm. **B** Assessment of the proportion of axons myelinated in each region at 3, 8 and 26 weeks of age. No significant difference was observed in the proportion of axons myelinated between *Gpr62*^WT/WT^ and *Gpr62*^KO/KO^ mice at any age. **C** G-ratio analysis of myelin thickness in the 3 and 8-week old optic nerve of wild-types and knockouts indicates normal myelin thickness in the *Gpr62*^KO/KO^ animals. Quantified in (**D**) based on *n* = 3 mice per condition, 200–300 myelinated axons assessed per animal

To determine whether myelin ultrastructure may be altered by the absence of Gpr62, we performed transmission electron microscopy analysis of myelin in the optic nerve, lateral white matter of the spinal cord and corpus callosum of *Gpr62*^WT/WT^ and *Gpr62*^KO/KO^ mice at 3 weeks, 8 weeks and 26 weeks of age. Both *Gpr62*^WT/WT^ and *Gpr62*^KO/KO^ mice showed the expected age-related increase in myelination of both tracts with age, with the corpus callosum myelinating later than the early-myelinating optic nerve (Fig. 6A, B). At no age assessed did the *Gpr62*^KO/KO^ mice show an altered proportion of axons myelinated relative to their *Gpr62*^WT/WT^ littermates in any of the three CNS regions assessed (*n* = 3, *p* > 0.05 for all comparisons). To investigate whether signaling through Gpr62 may regulate myelin thickness, we performed a g-ratio analysis in the optic nerve at both 3 and 8 weeks of age (Fig. 6C, D). The myelin was of an appropriate thickness in the *Gpr62*^KO/KO^ mice at both ages when g-ratios were pooled and compared across all axonal diameters (WT: 0.76 ± 0.01, KO: 0.75 ± 0.01 at 3 weeks of age and WT: 0.76 ± 0.01, KO: 0.75 ± 0.01 at 8 weeks of age, *p* > 0.05). Similarly, no significant difference was found in the average g-ratios between genotypes when small (< 0.6 μm), medium (0.6–0.9 μm) and large (> 0.9 μm) diameter axons were analyzed separately (Fig. 6C and data not shown). Although these results do not strictly preclude more subtle alterations to myelination in the absence of Gpr62 (such as changes to the populations of axons myelinated or changes in internode length), it would appear that Gpr62 is overall dispensable for CNS myelination in mice, at least out to 6 months of age.

## Discussion

Gpr62 is an orphan GPCR that shows relatively restricted expression to the brain and testes, with expression in the CNS being highly enriched in mature oligodendrocytes (Fig. 3, [24–26]). Given this preferential expression by oligodendrocytes we sought to assess the role of Gpr62 in myelination; oligodendrocytes’ best characterized role. The expression of *Gpr62* is induced late in oligodendrocyte differentiation, mirroring mature markers such as Nkx6–2, MOBP and MOG [24, 35, 36] and likely occurring after the initial stages of axonal ensheathment are complete. As such, a role for Gpr62 in regulating OPC dynamics or the initial stages of oligodendrocyte differentiation was unlikely, pointing towards potential roles in later processes such as myelin remodeling or oligodendrocyte survival. Nevertheless, we found no evidence for overall changes in myelination or oligodendrocyte numbers in *Gpr62* knockout mice. The density of mature oligodendrocytes was unaltered at both early stages of development and into adulthood. Overall levels of CNS myelination and markers of the node of Ranvier were not detectably altered by the absence of Gpr62. Similarly, no changes were detected in the proportion of CNS axons myelinated within the optic nerves, corpus callosi or spinal cord white matter or the thickness of myelin in *Gpr62*^KO/KO^ animals relative to their wild-type littermates. Mutants for some myelin proteins such as CNP only develop degenerative phenotypes later in life [43], however, the *Gpr62*^KO/KO^ mice were viable to at least 12 months of age with no overt motor phenotypes (data not shown), making a late-onset phenotype unlikely. These findings indicate that in spite of the expression of Gpr62 by oligodendrocytes and apparent adaxonal localization, it is dispensable for CNS myelination.

A recent characterization of an independently generated *Gpr62* knockout line failed to detect any phenotype in spermatogenesis, in spite of *Gpr62* expression in the testes [25]. This lack of phenotype was hypothesized to be due to likely redundancy with the closely related Gpr61. Given that the mRNA expression of *Gpr61* is at least an order of magnitude lower in mature oligodendrocytes than *Gpr62* [24, 35] and that we saw no up-regulation of *Gpr61* transcripts in the Gpr62 knockout mice by qRT-PCR, it seems less likely that the lack of an overt myelin phenotype is due to redundancy with Gpr61 in the oligodendrocyte lineage. This leaves open the possibility that other oligodendrocyte GPCRs operating through overlapping signaling pathways to Gpr62 may be able to compensate for its absence, however. Gpr62 can constitutively activate cAMP in heterologous cell assays and lacks canonical DRY and BBXXB motifs [25]. Constitutive activation of the Gs/cAMP and Gq/IP1 pathways by Gpr62 has also been reported with overexpression in 293 T cells [28], suggesting that Gpr62 likely acts through cAMP and/or calcium mediated pathways. If this also holds true for Gpr62 signaling within oligodendrocytes, it would act via distinct or opposing pathways to Gpr17 and Gpr37, both of which primarily signal through G_i/o_ proteins to limit cAMP and ERK signaling [19, 44, 45]. Given that cAMP is upstream of MAPK signaling, a pathway which positively regulates myelin formation by oligodendrocytes [46–50], it is conceivable that local expression and signaling from Gpr62 at myelin sheaths would positively regulate myelin thickness or extension. Nevertheless, we did not observe detectable changes in myelin thickness in the Gpr62 knockout mice either. Whether other receptors such as FGFR2, which is present in the non-compact myelin [51], could compensate for absence of Gpr62 remains to be seen.

Strikingly, we observed strong co-localization of a virally expressed Gpr62-Flag construct with MAG in myelinating oligodendrocytes in vivo. Very little expression of the tagged construct was present at the soma or abaxonal myelin surface of transduced oligodendrocytes, indicating that the protein was not evenly distributed across the plasma membrane of transduced cells. This suggests that Gpr62 contains motifs that direct its localization to the adaxonal myelin compartment, where it would be well placed to interact with axonal ligands. Gpr62’s current orphan status presents a substantial challenge to understanding what these interactions would be. Although Gpr62 has been classed as a likely biogenic receptor, a group that includes many receptors for neurotransmitters and other signaling molecules [27], at present interactions between amines and Gpr62 have not been demonstrated. Intriguingly, Gpr62 was recently identified in a screen for proteins that interact with C1q on human neural stem cells [52]. Although C1q is primarily considered to be a key component of the classical complement pathway in immunity [53], neurons also express C1q where it has been shown to play a key role in synapse refinement and axonal outgrowth [54–56] Whether oligodendroglial Gpr62 could interact with axonally expressed C1q will be an important question to address. A recent screen for small compound GPCR ligands identified several compounds that enhance Gpr62 activity, including 3-hydroxy-1,2-dimethyl-4(1H)-pyridone and Bifemelane [57]. An important future direction will be to establish whether these compounds alter oligodendrocyte behavior, and, if so, whether efficacy is lost in *Gpr62*^KO/KO^ mice, to better understand Gpr62’s normal role.

## Conclusions

This study sought to identify the role of the orphan GPCR Gpr62 in CNS myelination. We find that a GPR62-Flag construct expressed in oligodendrocytes in vivo preferentially co-localizes with MAG at the adaxonal myelin. This, together with the expression of *Gpr62* relatively late in oligodendrocyte differentiation, strongly suggests that endogenous Gpr62 might be involved in aspects of axo-myelinic signaling at the mature myelin sheath. Nevertheless, Gpr62 appears to be broadly dispensable for CNS myelination and oligodendrocyte maintenance, indicating it either mediates very subtle aspects of myelination or other aspects of oligodendrocyte behaviour or is redundant with other signaling molecules. Future gain-of-function experiments are likely to be critical for elucidating the function of this GPCR.

## Data Availability

All mouse lines, plasmids and other reagents are available upon request to the corresponding author.

## Abbreviations

AAV: Adeno-associated virus
Caspr: Contactin-associated protein
CNS: Central nervous system
Gpr62: G protein-coupled receptor 62
MAG: Myelin-associated glycoprotein
MBP: Myelin basic protein
Nav1.6: Voltage gated sodium channel 1.6
OPC: Oligodendrocyte progenitor cell
PLP: Proteolipid protein
UTR: Untranslated region
